# A Monolithic Electrochemical Micro Seismic Sensor Capable of Monitoring Three-Dimensional Vibrations

**DOI:** 10.3390/s18041047

**Published:** 2018-03-31

**Authors:** Lianhong Chen, Zhenyuan Sun, Guanglei Li, Deyong Chen, Junbo Wang, Jian Chen

**Affiliations:** 1 State Key Laboratory of Transducer Technology, Institute of Electronics, Chinese Academy of Sciences, 100190 Beijing, China; chenlianhong15@mails.ucas.ac.cn (L.C.); sunzhenyuan@mail.tsinghua.edu.cn (Z.S.); liguanglei13@mails.ucas.ac.cn or wkliguanglei@163.com (G.L.); jbwang@mail.ie.ac.cn (J.W.); chenjian@mail.ie.ac.cn (J.C.); 2 School of Electronic, Electrical and Communication Engineering, Chinese Academy of Sciences, 100190 Beijing, China

**Keywords:** monolithic electrochemical seismic sensor, three-dimensional vibrations monitoring, decoupling mechanism

## Abstract

A monolithic electrochemical micro seismic sensor capable of monitoring three-axial vibrations was proposed in this paper. The proposed micro sensor mainly consisted of four sensing units interconnected within flow channels and by interpreting the voltage outputs of the sensing units, vibrations with arbitrary directions can be quantified. The proposed seismic sensors are fabricated based on MEMS technologies and characterized, which produced sensitivities along *x*, *y*, and *z* axes as 2473.2 ± 184.5 V/(m/s), 2261.7 ± 119.6 V/(m/s), and 3480.7 ± 417.2 V/(m/s) at 30 Hz. In addition, the vibrations in *x*-*y*, *x*-*z*, and *y*-*z* planes were applied to the developed seismic sensors, leading to comparable monitoring results after decoupling calculations with the input velocities. Furthermore, the results have shown its feasibilities for seismic data recording.

## 1. Introduction

Seismic sensors capable of sensing the seismic motions of the earth are considered as key components in the field of seismology [1], such as geophysical exploration [2,3] and seismic monitoring [4,5,6]. Especially in some applications, like structure damage detection [7,8,9,10,11], some automated sensors with smart materials are used [12,13]. According to the differences in operation principles, the conventional seismic sensors are mainly divided into variable capacitance seismic sensors [14,15], fiber-optic sensors [16,17], micro-electromechanical system accelerometers [18,19], and electrochemical seismic sensors [20,21]. Due to the use of liquid proof masses, electrochemical seismic sensors are featured with high sensitivities and low noise levels in the low-frequency domain, which is successfully used in self-contained broadband bottom seismographs.

However, in some field where the monitoring of three-dimensional motion actually is widely required from the perspective of applications (e.g., earthquake early warning system [22], seismic data recording system [23] and furthermore measuring shear stress in aerodynamics structures [24]), the monitoring of three-dimensional motions based on electrochemical approach, which is usually orthogonally mounted by three unidirectional seismic sensors, suffers from key limitations of bulky structures and high cost [21].

To deal with this issue, a monolithic electrochemical seismic sensor was proposed, which consisted of the acrylic glass housing, two membranes, an electrolyte solution, and four sensing units, enabling the monitoring of three-dimensional vibration signals. More specifically, the sensing units were located at the centers of the flow channels inside the glass housing and were connected with each other by electrolyte solutions (KI and $I_{2}$), which were filled into the space between the glass housing and the membranes. On top of the housing, four sensing units were separated by a separation plate.

## 2. Structure and Operating Principle

**Figure 1:**
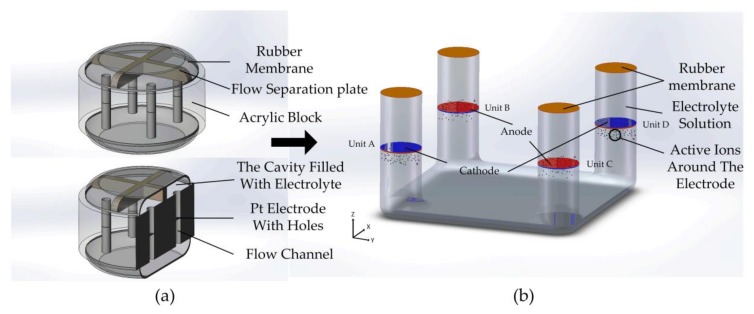
(**a**) Overall views and cross-section views of the proposed device with four flow channels and sensing units inside them. (**b**) The simplified schematic of the liquid part in the proposed device.

The proposed structure of monolithic seismic sensor, including the overall schematic, the cross-section in *x* and *y* axial direction and the schematic of sensing units in the flow channel were shown as Figure 1. The external vibration, which is usually represented as the velocity form, was applied to the device resulting in the convection of the liquid with the support of the rubber membrane. The sensing units in channels detected the change of active ions caused by the convection and then output the electric information.

In order to improve the performance in sensitivities and reliabilities of the proposed devices, the cross-flow separation plates shown in Figure 1a were included to limit the flow of electrolyte solutions. The separation plates divided the liquid masses into four parts in four channels, which were connected with each other through the bottom of the device. The liquid part in this modified structure was simplified ias Figure 1b.

More specially, the sensing mechanism can be explained by a mechanical part and an electrochemical part. The mechanical part, which consists of an acrylic glass block, rubber membranes and a liquid inertial mass, transfers the external vibrations to the velocities of the liquid mass inside the channel based on the restoring force generated by the membranes.

Due to the effects of the external vibrations in membranes and the exist of the viscous forces in the liquid, the convection of the electrolyte solution was generated. According to the Newton’s Second Law of Motion, the characteristic of the mechanical part is expressed as follows.

(1) $$m\frac{d^{2}V}{{\mathrm{dt}}^{2}}\frac{1}{S_{c}}+R_{h}S_{c}\frac{\mathrm{dV}}{\mathrm{dt}}+\mathrm{kV}=-m\frac{{\mathrm{dv}}_{\mathrm{ex}}}{\mathrm{dt}},$$

where $v_{\mathrm{ex}}$ represents the amplitudes of the external vibration velocity; $S_{c}$ represents the cross sectional area of the channels; $R_{h}$ represents the flow resistance coefficient and k represents the elastic coefficient of the rubber membrane. The transfer function of mechanical part is solved as follow.

(2) $$\left|H_{1}\left(\omega \right)\right|=\left|\frac{v_{\mathrm{in}}}{v_{\mathrm{ex}}}\right|=\frac{\omega^{2}}{\sqrt{{\left(\omega^{2}-\omega_{0}^{2}\right)}^{2}+R_{e}^{2}\omega^{2}}},$$

where $\omega_{0}=\sqrt{\frac{k}{m}}$ represents the resonant frequency of the device and $R_{e}=\frac{R_{h}S_{c}^{2}}{m}$ represents the equivalent damping [25]. As presented in (2), with the same frequency and channel geometries, the output velocity of the liquid and the input vibration velocity remain linear.

The electrochemical part, including an electrolyte solution and sensing units, ensured that the motions of the liquid mass in channels can be transferred into the electrical outputs completely. The sensing unit composed of an insulation silicon substrate layer and a pair of planar platinum electrodes fabricated on the both surfaces of the substrate was fixed transversely in the center of channels, as shown in Figure 1a. The layouts of the anodes and the cathodes of the sensing units in channel B and C were opposite to that in channel A and D. The voltage drops between the anodes and the cathodes in the sensing units were set to 0.3 V. With the application of an electric potential, electrochemical reactions $3I^{-}-2e^{-}\to I_{3}{}^{-}$ and $I_{3}{}^{-}+2e^{-}\to 3I^{-}$ [24] occur on the anodes and cathodes, respectively.

The electrode currents were generated by the ionic flux on the electrodes. According to the Faraday’s Law, cathode output currents were presented as follow.

(3) $$I_{o}=\mathrm{nF}\int Jn\mathrm{ds},$$

where n = 1 represents the number of the exchanged electronics in the reaction; F = 96,500 represents the Faraday constant; n represents the unit normal vectors on the cathode; s represents the surface area of the cathode. Besides, the Nernst-Plank equation, Fick Laws, Navier-Stocks equation, and Butler-Volmer equation describe the transfer in the electrochemical part [25]. With the linear simplification of the boundary conditions, the transfer function of the electrochemical part can be expressed as:

(4) $$\left|H_{2}\left(\omega \right)\right|=\left|\frac{I_{o}}{v_{\mathrm{in}}}\right|=\frac{C}{\sqrt{1+{\left(\frac{\omega }{\omega_{D}}\right)}^{2}}},$$

where C is the transfer constant, which depends on the device; $\omega_{D}$ represents the diffusion frequency, which is related to the diffusion coefficient of the liquid. Based on an operational amplifier and a decoupling circuit, the cathode currents can be changed to voltages, where the amplitudes of voltages are proportional to the input vibration velocities.

Without environmental vibrations, as shown in Figure 1b, a stable concentration gradient of $I_{3}{}^{-}$, the active ions in the electrochemical reactions due to their far lower concentrations than $I^{-}$ [25], forms between anodes and cathodes, resulting in an equilibrium stage where the cathode currents through every unit remain equal. Due to the exists of differential calculations in the decoupling circuit, the output voltage is zero.

In response to an external vibration along different axis, the mechanisms are different. For example, in case of a seismic vibration in the x axis, the output voltage of the direction vibration ($u_{x}$) is represented as follow.

(5) $$u_{x}=\left(u_{A}+u_{B}\right)-\left(u_{C}+u_{D}\right)=u_{A}-u_{C}+u_{B}-u_{D}$$

where $u_{x}$ represents the final output voltage in response to the vibration along the axis and $u_{A},\;u_{B},\;u_{C}$, and $u_{D}$ represent the outputs of sensing units in the channel A, B, C, and D.

**Figure 2:**
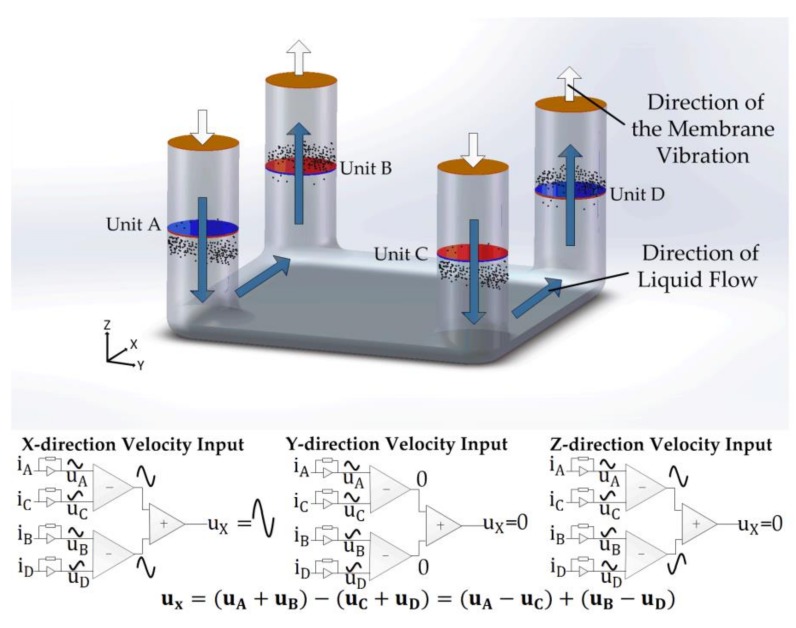
Sensing mechanism illustrations and schematics of fluid flowing mechanism in response to acceleration along *x*-axis. Blue arrows represent the direction of liquid flow due to the convection caused by the external vibration while white arrows represent the direction of the membrane vibration. $i_{A},\;i_{B},\;i_{C}$, and $i_{D}$ represent the cathode currents in sensing unit A, B, C, D, respectively.

The operating mechanism under the condition of *x*-axis vibration is presented in Figure 2. Due to the limit of the separation plate and the membranes, the electrolyte solution flows as shown in Figure 2, resulting in an opposite change of liquid flow in channel A and channel B. but due to the opposite cathode-anode layout, the active ions both moved from the cathode to the anode in each channel in A and B, producing comparable current outputs.

Thus, $u_{A}$ and $u_{B}$ should be added together to double the output. The same phenomenon takes place in channels C and D. Owing to the opposite cathode-anode layout between the sensing units in channel A and C, B and D, the amplitudes of formula $(u_{A}+u_{B}$) are opposite to that of ($u_{c}+u_{D})$ and, thus, the final output voltage is formed by the difference of $(u_{A}+u_{B}$) and ($u_{c}+u_{D})$.

Equation (5) also ensures that $u_{x}$ is not affected by the vibrations in other axes. For example, in response to the *y*-vibration, the outputs in $u_{A}$ and $u_{C}$ caused by the corresponding motion of the solution were positive while the outputs in $u_{B}$ and $u_{D}$ were negative in the same half period. Through the equation ($u_{A}-u_{C}$) and ($u_{B}-u_{D}$), the same outputs would be offset. In the same way, for cases of *z* vibrations along the axis, the outputs of sensing units in channels A and D, B and C are comparable, canceling the output of u**_x_**.

The output voltage in response to the *y* direction vibration ($u_{y}$) is represented as:

(6) $$u_{y}=\left(u_{A}+u_{C}\right)-\left(u_{B}+u_{D}\right)=u_{A}-u_{B}+u_{C}-u_{D}$$

where $u_{y}$ represents the final output voltage in response to the vibration along the *y* axis.

**Figure 3:**
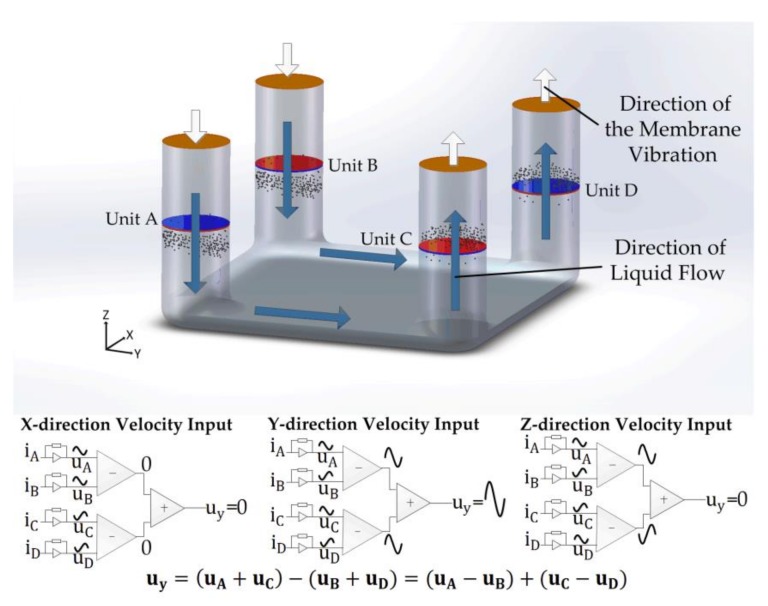
Sensing mechanism illustrations and schematics of fluid flowing mechanism in response to acceleration along *y*-axis. Blue arrows represent the direction of liquid flow due to the convection caused by the external vibration while white arrows represent the direction of the membrane vibration. $i_{A},\;i_{B},\;i_{C}$, and $i_{D}$ represent the cathode currents in sensing unit A, B, C, D, respectively.

The operating principle can be explained in Figure 3. Similarly with *x*-axis vibration, an opposite change of active ion concentrations in channel A and channel C was generated. $u_{A}$ and $u_{C}$, $u_{B}$ and $u_{D}$ should be added together to double the output and the final output can be expressed as the difference of $(u_{A}+u_{C}$) and ($u_{B}+u_{D})$ as represented in Equation (6). In the same way with Equation (5), the comparable output caused by *x*-vibration and *z*-vibration can be cancelled through the decoupling, $U_{y}$ was zero.

**Figure 4:**
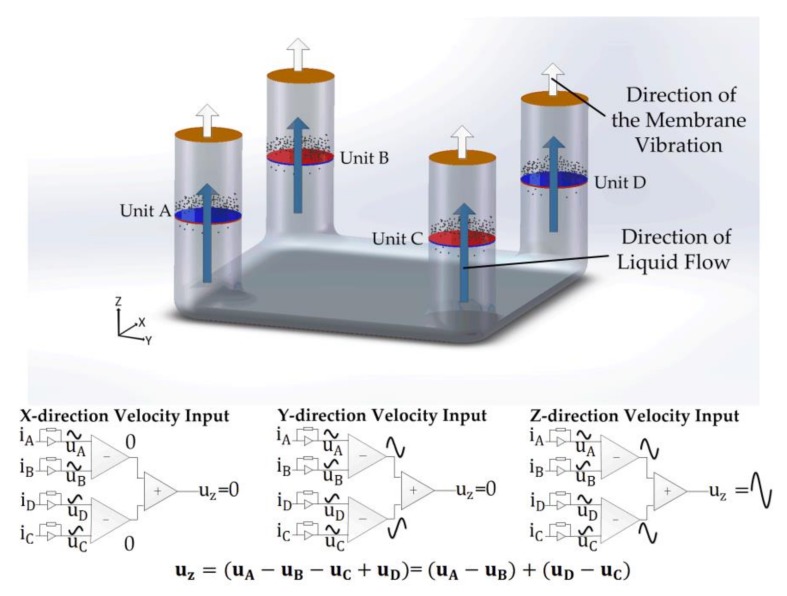
Sensing mechanism illustrations and schematics of fluid flowing mechanism in response to acceleration along *z*-axis. Blue arrows represent the direction of liquid flow due to the convection caused by the external vibration while white arrows represent the direction of the membrane vibration. $i_{A},\;i_{B},\;i_{C}$, and $i_{D}$ represent the cathode currents in sensing unit A, B, C, D, respectively.

The operating mechanism under the condition of *z*-axis vibration is shown in Figure 4. When a *z*-axial vibration is applied, the electrolyte solution moves along the direction as arrows described, leading to identical movements in channels of A, B, C, and D. Thus, due to the opposite cathode-anode layout in sensing units in channels B and C, the output voltage of the *z*-direction vibration ($u_{z}$) is represented as:

(7) $$u_{z}=u_{A}-u_{B}-u_{C}+u_{D}=u_{A}-u_{B}+u_{D}-u_{C}$$

Again, in response to the seismic vibrations from other axes including both *x* and *y* directions, there is no output and $u_{z}$ is always zero. Thus, Equation (7) ensures that $u_{z}$ is not affected by the vibrations in the other axes.

## 3. Fabrication

**Figure 5:**
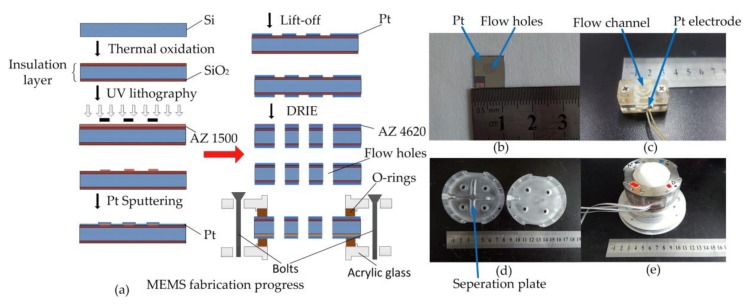
(**a**) The fabrication progress of the electrodes in sensing units, including thermal oxidation, Pt sputtering, lift-off and deep reaction ion etching. (**b**) The electrode fabricated by MEMS technology with a lot of flow holes. (**c**) The sensing unit composed by electrodes and flow channels. (**d**) The acrylic glass housing with separation plate and flow channels. (**e**) The assembled device.

Figure 5a showed the fabrication progress of the electrodes in sensing units with Micro-electromechanical Systems (MEMS) technology. Firstly, the insulating spacers based on 130 μm thick silicon wafer were formed by the 600 nm thick silicon oxide layer on both sides of the wafer substrate. Then the AZ1500 photoresist was spin-coated on one side of the wafer. Through the lithography progress, the arrays of holes were patterned by UV lithography after the soft bake of the photoresist. A 120 nm platinum electrode was sputtered on one side of the wafer. After the lift-off progress, the area of holes was exposed. The electrode on the other side of the wafer was conducted in the same way and two totally same electrodes were formed. Next, DRIE (deep reaction ion etching) was used to form the channels in the wafer with AZ4620 photoresist pre-coated on the front side of the wafer to protect the electrodes from being etched in DRIE progress.

Then the electrode chips were compressed mechanically by the unit glass housing and two O-shaped rubber rings in between with bolts. The O-rings were easily deformable so that the channels described in Figure 5a can be sealed completely instead of the use of any kinds of adhesive.

The pictures of electrode chips with flow holes after MEMS progress and the sensing unit were shown as Figure 5b,c.

The sensing units were immobilized in an acrylic glass housing (see Figure 5d) with the alignment of the holes in the sensing units and the glass housing, forming flow channels. The membranes covered the top and bottom surfaces of the housing to form a sealed cavity by mechanical compressing. The electrolyte solution was then injected into the cavity through a liquid valve in the housing. The assembled device was shown in Figure 5e.

## 4. Results

The performances of the three-dimensional devices were measured by a vertical platform for the characterization of *x*- and *y*-axial vibrations and a horizontal platform to characterize z-axial responses. More specifically, a 4808-type vibration exciter manufactured by B & K in 22820 Savi Ranch Parkway, Yorba Linda, CA, USA [25] was included in the vertical test and a low-frequency acceleration calibration system fabricated by Dongling Technologies in Suzhou city, Zhejiang province, China was used in the horizontal characterization.

**Figure 6:**
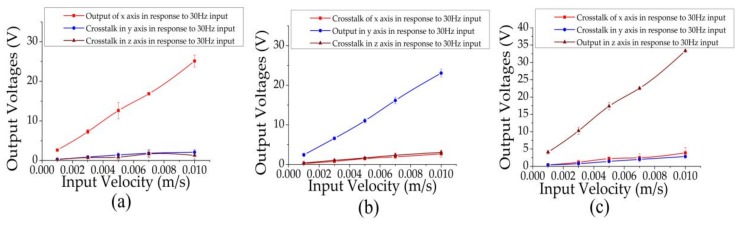
Independent output voltages along *x*, *y*, and *z* axes in response to the input vibration at 30 Hz along *x* (**a**), *y* (**b**), and *z* axes (**c**), where huge differences between the input axial outputs and the others proved that when the vibration along an axis input, the crosstalk caused by the other axis can be ignored.

To identify both sensitivities and cross-talks of the proposed monolithic seismic sensor in response to three-dimensional vibrations, experiments with single inputs at the *x*, *y*, or *z* axes were conducted (see Figure 6).

Figure 6a presented the output voltages of three axes in response to *x*-axis vibrations. The sensitivity at the *x* axis at 30 Hz was characterized as 2473.23 ± 184.55 V/(m/s). Meanwhile, in response to *x*-direction vibrations, the voltage outputs at *y* and *z* directions were much lower compared to the values of the *x*-direction voltages. The ratios of output and the crosstalk $u_{x}$/$u_{y}$ and $u_{x}$/$u_{z}$ were 7.65 and 8.66 at 30 Hz. The unwanted output voltages at the *y* and *z* directions were due to the mismatched electrodes in micro-fabrication, which cannot be avoided entirely. However, this issue can be to an extent addressed by accurately controlling the fabrication and packaging steps to decrease potential geometrical differences among sensing units.

Figure 6b showed the voltage outputs of three axes in response to a vibration along the *y* axis. The sensitivity at the *y* axis at 30 Hz was quantified as 2261.71 ± 119.68 V/(m/s), which was comparable to the sensitivity of the *x* axis when *x*-directional vibrations were applied due to the axisymmetric structure in the *x-y* plane. The ratios of output and the crosstalk $u_{y}$/$u_{x}$ and $u_{y}$/$u_{z}$ were quantified as 8.34 and 7.27 at 30 Hz.

As shown in Figure 6c, the sensitivity of the *z*-axis in response to *z* vibration inputs was quantified as 3480.71 ± 417.20 V/(m/s) at 30 Hz, which was higher than the corresponding sensitivities in the *x* and *y* axes. It was speculated that due to the effect of the membranes, the motion of the liquid under the membranes, which was considered as the part with the largest velocity caused by the external horizontal vibration, cannot convert the horizontal velocity into the channel-direction velocity completely. Thus, external vibrations at the *z* direction without the energy loss in velocity conversion can lead to higher voltage outputs in comparison to *x* and *y* directional vibrations. The ratios of output and the crosstalk $u_{z}$/$u_{x}$ and $u_{z}$/$u_{y}$ were quantified as 8.84 and 11.57 at 30 Hz.

**Figure 7:**
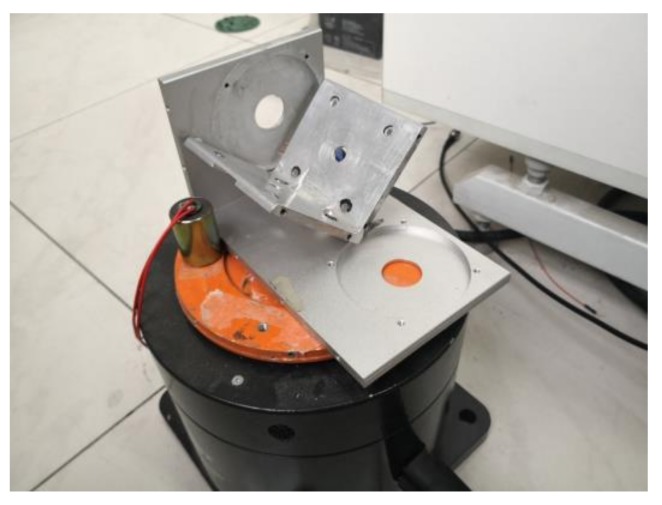
The schematic of the home-developed framework.

Furthermore, the outputs of the devices in response to applied inputs at the angles of 45° within the *x*-*y*, *x*-*z*, and *y*-*z* planes were collected. Note that the inputs were applied by the vibration exciter and a home-developed framework, as shown in Figure 7. The angle was strictly confirmed with angular measuring tools.

**Figure 8:**
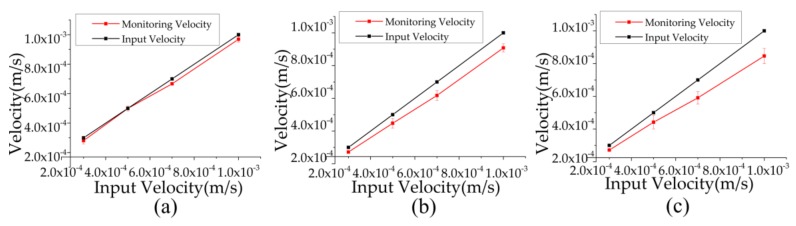
The comparisons of the monitoring velocity based on the decoupling mechanism and the actual input velocity in response to the vibration at the direction of 45-degree angle with the *x* axis in *x*-*y* plane (**a**), at a 45 degree angle with the *y* axis in *y*-*z* plane (**b**), and at a 45-degree angle with the *x* axis in *x*-*z* plane (**c**), respectively. The closed value between the monitoring results and the actual input proved the feasibility of the monolithic structure and the decoupling mechanism.

In response to an input within the *x*-*y* plane, the corresponding monitoring velocities calculated with the vector synthesis by the ratios of the axial outputs and the corresponding sensitivities in *x* and *y* axes, shown in Figure 6, were quantified as the function of input velocities (see Figure 8a).

As shown in Figure 8a, the slopes of the curves in response to the 45-degree angle inputs were quantified as 0.97, which were comparable with the slopes of the input curves, validating the feasibility of the monolithic device. In response to inputs within the *y*-*z* plane, the slopes of the monitoring curves in response to the 45-degree angle inputs were quantified as 0.90, as shown in Figure 8b. Similarly, in response to the 45-degree angle *x*-*z* plane input, the slopes of the monitoring curves in response to the 45-degree angle inputs were quantified as 0.87 (see Figure 8c). The comparable curves proved the proposed devices were capable of monitoring the vibrations in any directions.

In summary, no matter which direction the vibrations were input along, the voltage outputs by the monolithic device were close to the theoretical result, which proved the feasibility in measuring three-dimensional vibration through the decoupling from the mix input.

## 5. Conclusions

In conclusion, a MEMS based monolithic electrochemical seismic sensor, which can monitor three-dimensional vibrations, was demonstrated in this paper. Experimental results showed the prominent differences of output voltages between input axis and the other axes. The ratios of the outputs and the crosstalk of the other axes are about 10, which illustrated the great independence and the negligible crosstalk. In addition, at 30 Hz, the sensitivity at the *x* axis was characterized as 2473.23 ± 184.55 V/(m/s). *y* axis sensitivity was quantified as 2261.71 ± 119.68 V/(m/s), and *z* axis sensitivity can reach 3480.71 ± 417.20 V/(m/s).

The vibration information along three axes can be decoupled accurately from the mixed input in *x-y*, *x*-*z*, and *y*-*z* plane. The average ratios of the monitoring velocities and the input at three axes were calculated as 0.97, 0.90, 0.87, respectively and the feasibility of the decoupling calculation can be proved from it.
